# Supplementary material for: Immunoproteasome expression is associated with better prognosis and response to checkpoint therapies in melanoma
Source: Nat Commun. 2020 Feb 14;11:896. doi: 10.1038/s41467-020-14639-9 (PMC7021791; doi:10.1038/s41467-020-14639-9)
Supplement: Supplementary file 1 — Supplementary Information [file 41467_2020_14639_MOESM1_ESM.pdf]

Supplementary information

Immunoproteasome expression is associated with better prognosis and response to checkpoint therapies in melanoma

Kalaora, Lee et al.

Supplementary Notes

Supplementary Note 1: Correlation between IP subunit expression and IFNγ and T-cell related variables becomes lost in high purity tumors

As there are more factors in the tumor environment that may affect the IP expression levels observed in the tumor specimens we controlled their effect by checking the correlation of these factors and IP expression in different tumor purity levels. These factors are: 1. Presence of T-cells that are known to express high levels of IP. 2. Presence of APCs that are known to express high levels of IP. 3. The levels of IFNγ in the tumor that can induce the expression of IP in tumor cells (controlled here by the expression of genes that are known to be induced by IFNγ and described as "IFNγ signature"). First we see negative correlation between the estimated APCs and IP expression (**Supplementary Fig. 4**), suggesting that they do not affect the IP expression level observed in the samples. Then we checked the correlation between IP expression and the expression of IFNγ, IFNγ-related genes (signature genes that are known to be induced by IFNγ) or T-cell related genes in the subset of samples with different tumor purity levels. We observe that the correlation between IP subunits and IFNγ, IFNγ genes or T-cell related genes is lost in high purity tumors, while it remains significant in low purity tumors (**Supplementary Fig. 5**). This suggests that the IP subunit expression observed in the samples are not mere consequence of IFNγ release or T-cell infiltration, but rather expression of IP by the tumor cells independent of IFNγ release or T-cell infiltration.

Supplementary Figures

a

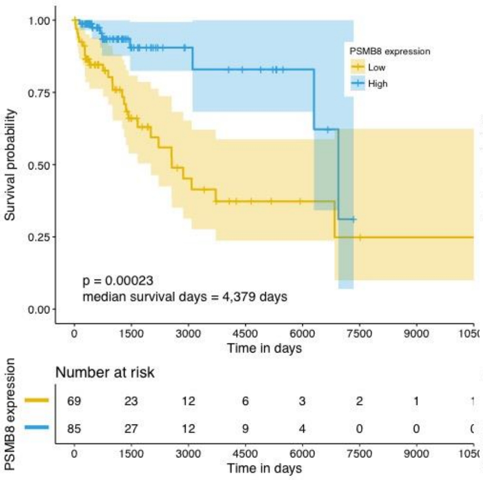

b

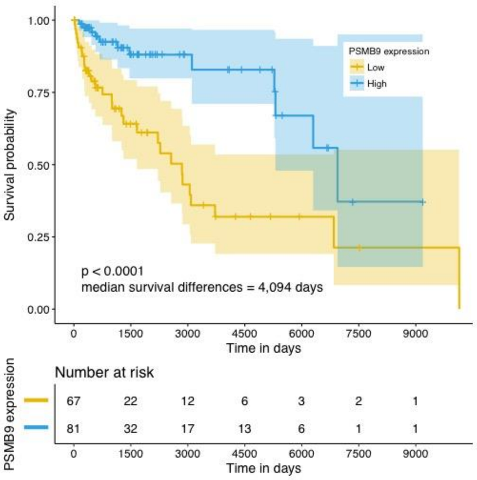

c

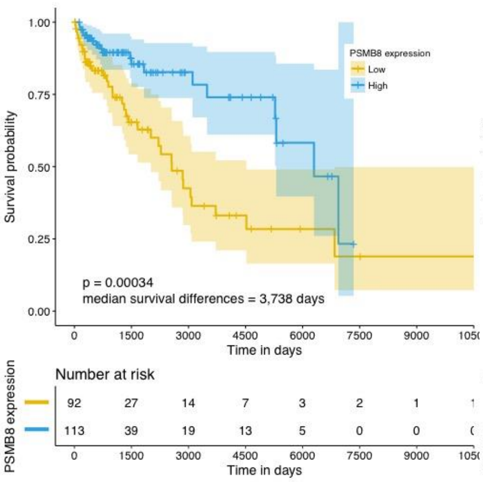

d

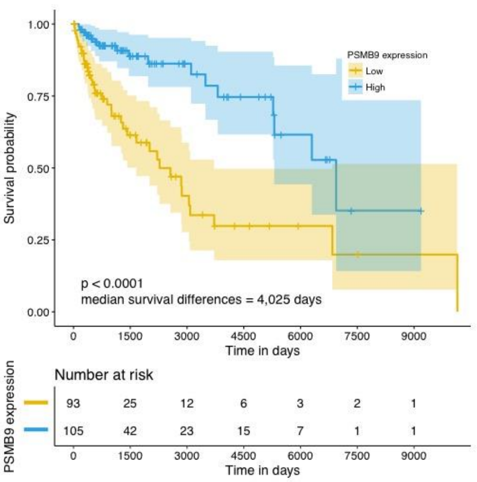

e

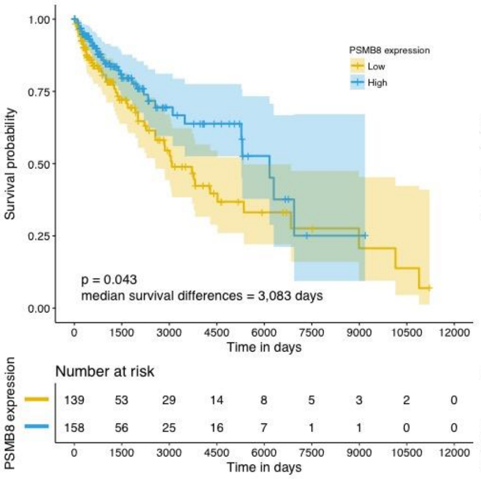

f

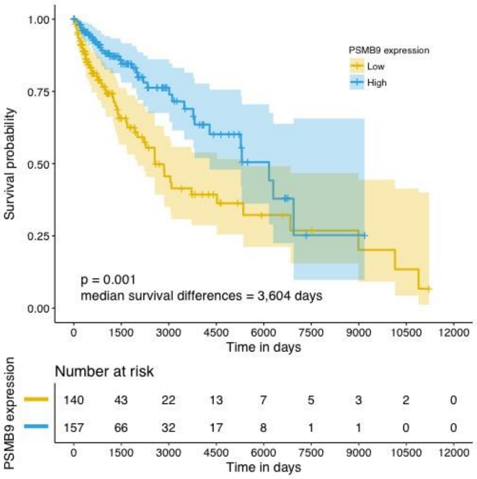

Supplementary Fig. 1. Kaplan Meier (KM) analysis of IP expression in TCGA melanoma patients with varying thresholds. The KM plots compares the survival of patients with high (blue) vs low (yellow) levels of (a, c, e) PSMB8 expression and (b, d, f) PSMB9 expression. High (low) levels of PSMB8/9 were determined based on top 25-percentile (bottom 25-percentile) for (a, b), top tertile (bottom tertile) for (c, d), and top 50-percentile (bottom 50-percentile) for (e, f). Two-sided logrank p-values are denoted in the figure, and the table on the bottom summarizes the patients at risk in different time points. The effect size was quantified by the difference in medial survival time of each patient group, leading to 4379, 4094, 3,738, 4,025, 3083, and 3604 from A to F, respectively.

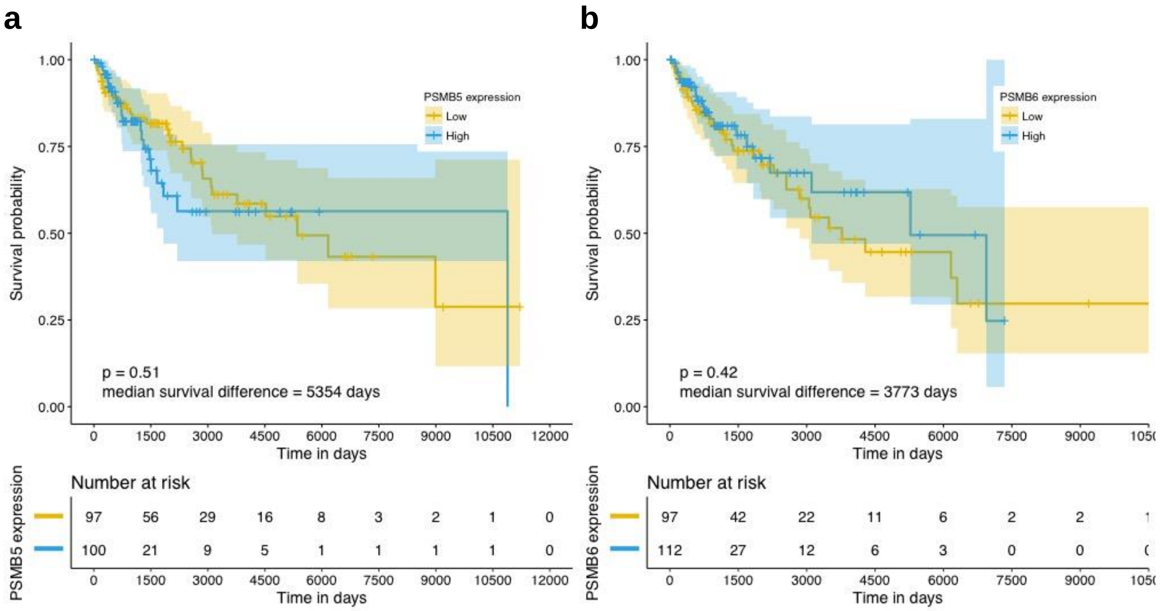

Supplementary Fig. 2. Kaplan Meier (KM) analysis of constitutive proteasome expression in TCGA melanoma patients. The KM plots compares the survival of patients with high (blue) vs low (yellow) levels of (a) PSMB5 and (b) PSMB6 expression. High or low levels of PSMB5/6 were determined based on top 33-percentile (bottom 33-percentile). Two-sided logrank p-values are denoted in the figure, and the table on the bottom summarizes the patients at risk in different time points.

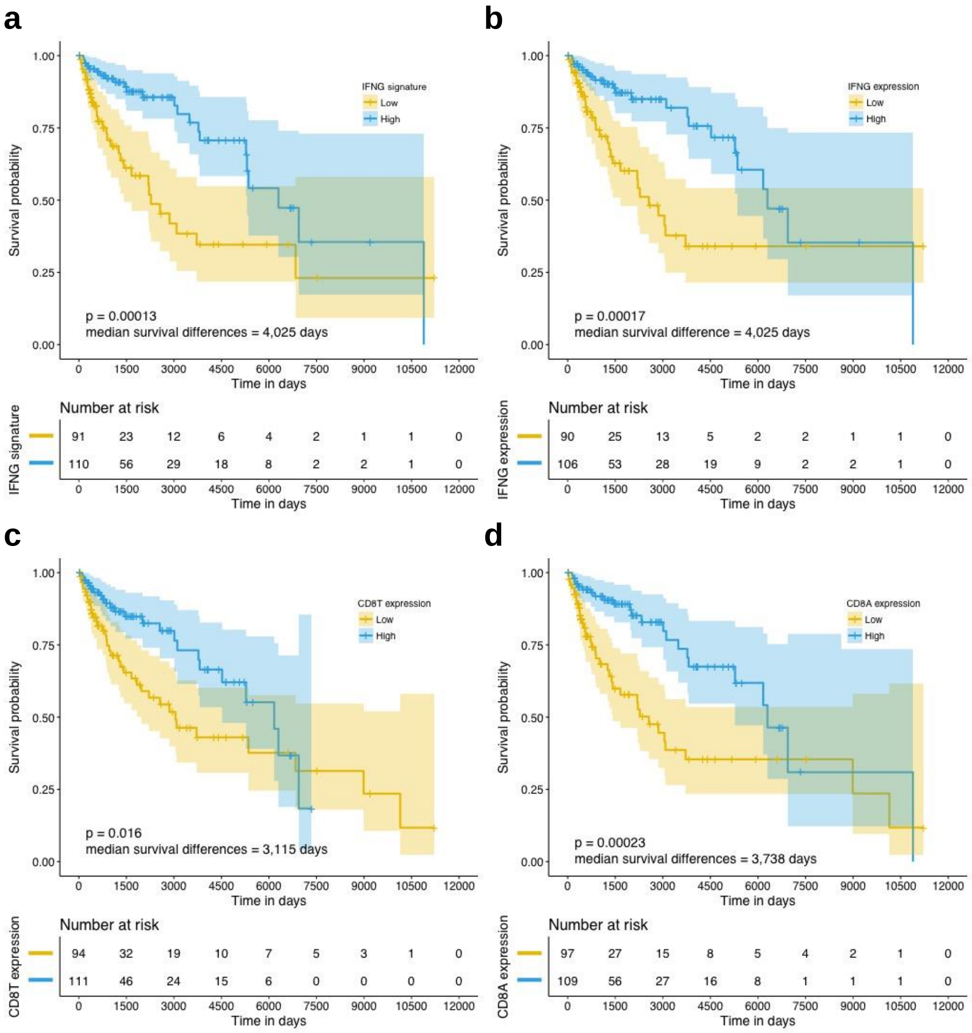

Supplementary Fig. 3. Comparative Kaplan Meier (KM) analysis of IFNγ and T-cell infiltration in TCGA melanoma patients. The KM plots compare the survival of patients with high (top tertile, blue) vs low (bottom tertile, yellow) levels of (a) IFNγ signature, (b) IFNγ expression, (c) estimated CD8+ T-cell abundance, and (d) CD8A expression. For each variable, the survival of patients with top tertile vs bottom tertile was compared using two-sided logrank test, and the p-values are denoted in the figure. The table on the bottom summarizes the patients at risk in different time points, and the median survival time differences are 4025, 3738, 3115, and 3738, respectively.

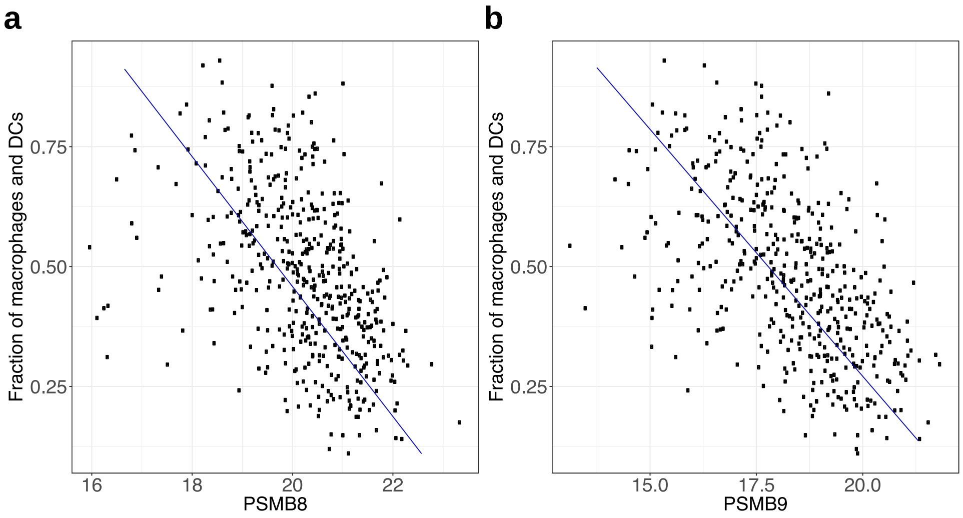

Supplementary Fig. 4. Correlation between IP and estimated abundance of APCs in TCGA melanoma. Y-axis shows the estimated abundance of APCs (dendritic cells and macrophage) and X-axis shows (a) PSMB8 and (b) PSMB9 expression in TCGA melanoma. Spearman correlation was -0.52 and -0.59 (P<2.2E-16), respectively.

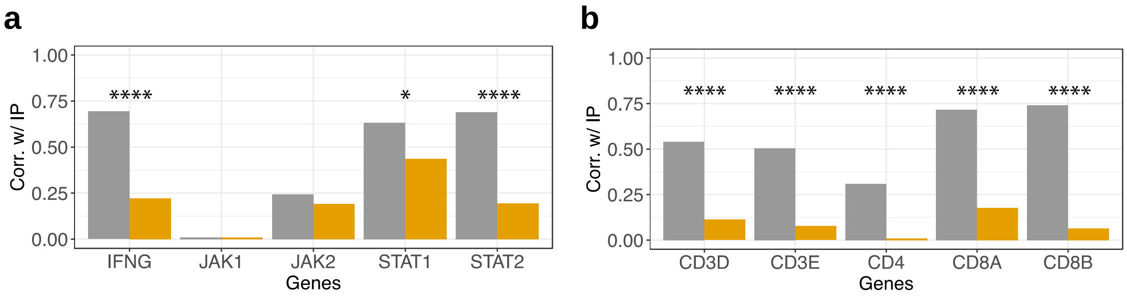

Supplementary Fig. 5. Correlation between IP and IFNγ related and T-cell related gene expression is lost in high purity tumors. Y-axis shows the correlation between the IP subunit expression and the expression of (a) IFNγ-related genes and (b) T-cell related genes in high (yellow; top 10-percentile) vs low (grey; bottom 10-percentile) tumors. The empirical p-value were calculated by randomly shuffling the sample labels and considering the probability that the difference in AUC of high purity tumors vs low purity tumors is reached by random chance. * denotes $P<0.05$, and **** denotes $P<0.0001$.

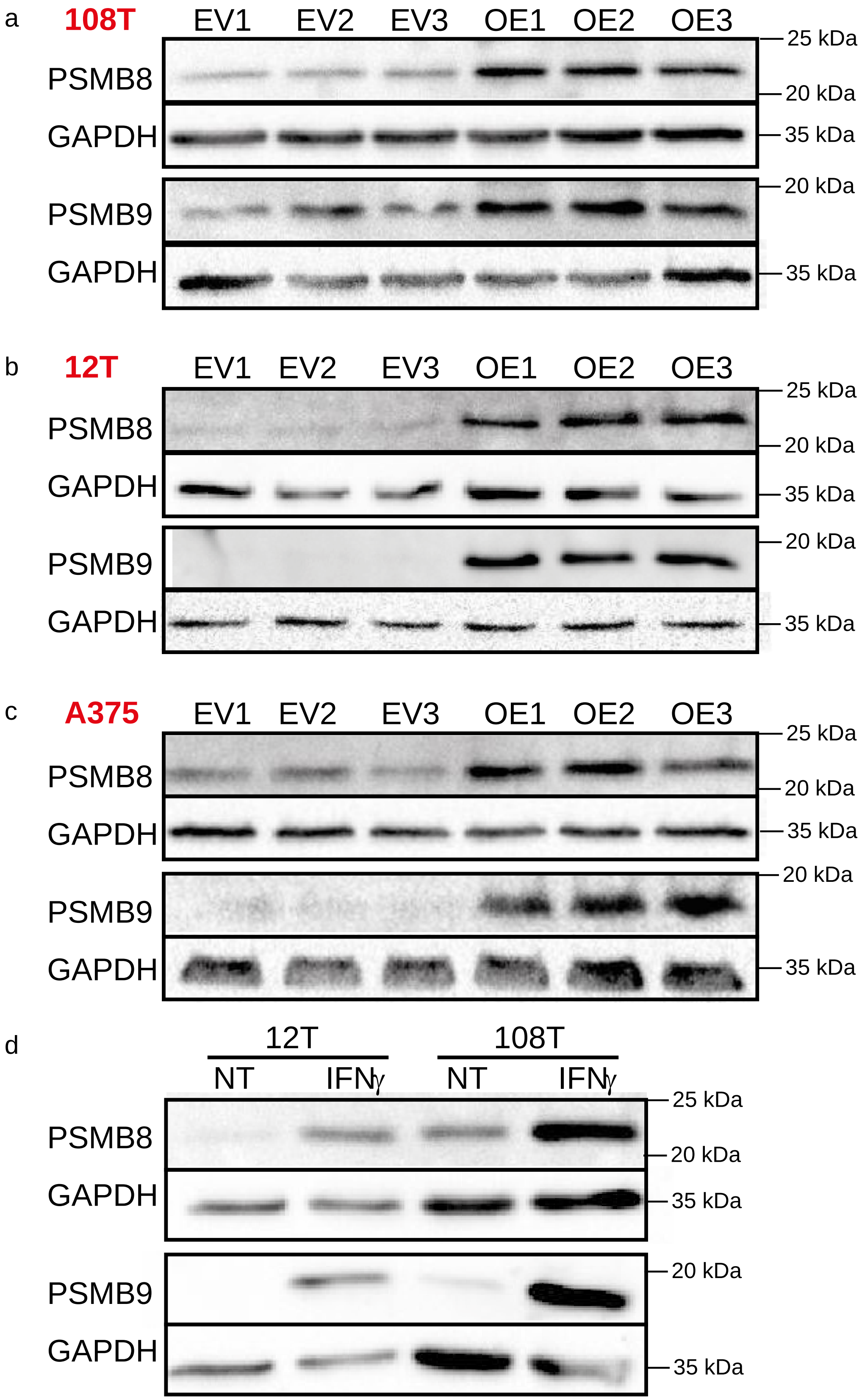
a
108T
EV1
EV2
EV3
OE1
OE2
OE3
PSMB8
GAPDH
PSMB9
GAPDH
25 kDa
20 kDa
35 kDa
20 kDa
35 kDa
b
12T
EV1
EV2
EV3
OE1
OE2
OE3
PSMB8
GAPDH
PSMB9
GAPDH
25 kDa
20 kDa
35 kDa
20 kDa
35 kDa
c
A375
EV1
EV2
EV3
OE1
OE2
OE3
PSMB8
GAPDH
PSMB9
GAPDH
25 kDa
20 kDa
35 kDa
20 kDa
35 kDa
d
12T
108T
NT
IFNγ
NT
IFNγ
PSMB8
GAPDH
PSMB9
GAPDH
25 kDa
20 kDa
35 kDa
20 kDa
35 kDa

Supplementary Fig. 6. IP subunits expression level in cells with stable overexpression or cells that were treated with IFNγ. (a-c) Western blot analysis of the expression of the IP subunits PSMB8 and PSMB9 in 108T, 12T and A375 cell lines that stably overexpress IP subunits (OE) or empty vector control (EV). Each cell line was infected independently three times to create three different cell cultures. GAPDH was used as loading control. (d) Western blot analysis of the expression of the IP subunits PSMB8 and PSMB9 in 108T and 12T cells that were treated with 250U/ml IFNγ for 48 hours (IFNγ) or not treated (NT). GAPDH was used as loading control. Figures are representative of n=2 repetitions that showed similar results. The uncropped blots are found in Source Data section.

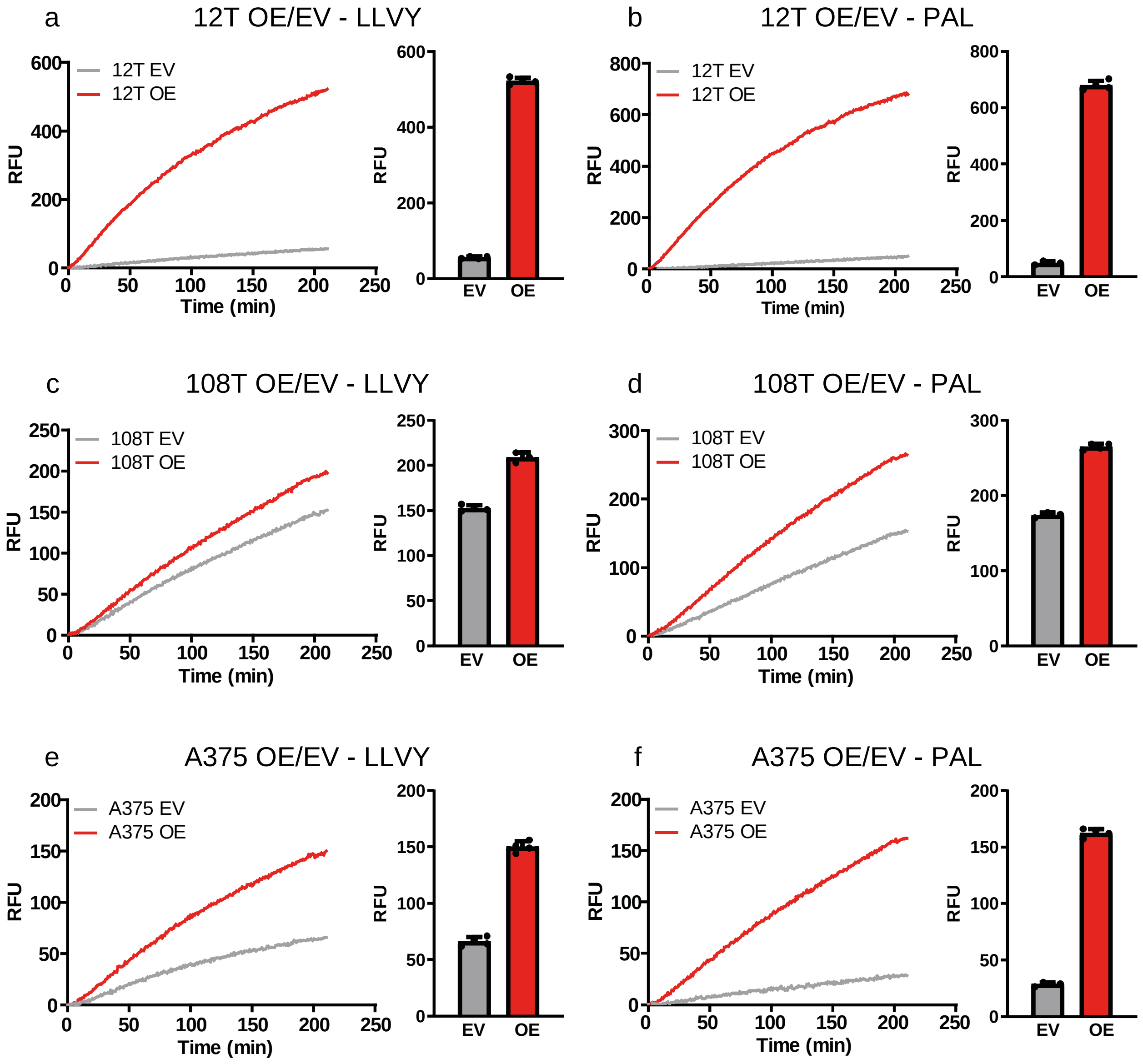

Supplementary Fig. 7. Immunoproteasome is more active in cells with overexpression. Cell lysates of immunoproteasome overexpression cells were incubated with Suc-LLVY-AMC (a, c and e) and Suc-PAL-AMC (b, d and f) peptides to test their PSMB5 and PSMB8 or their PSMB9 catalytic activity respectively, for 3.5 hours. Relative fluorescence unit (RFU) were calculated and plotted over time. RFU was calculated from n=4 biological replicates of the starting and end time points, and is indicated in the bar plot which represented as mean ± SD.

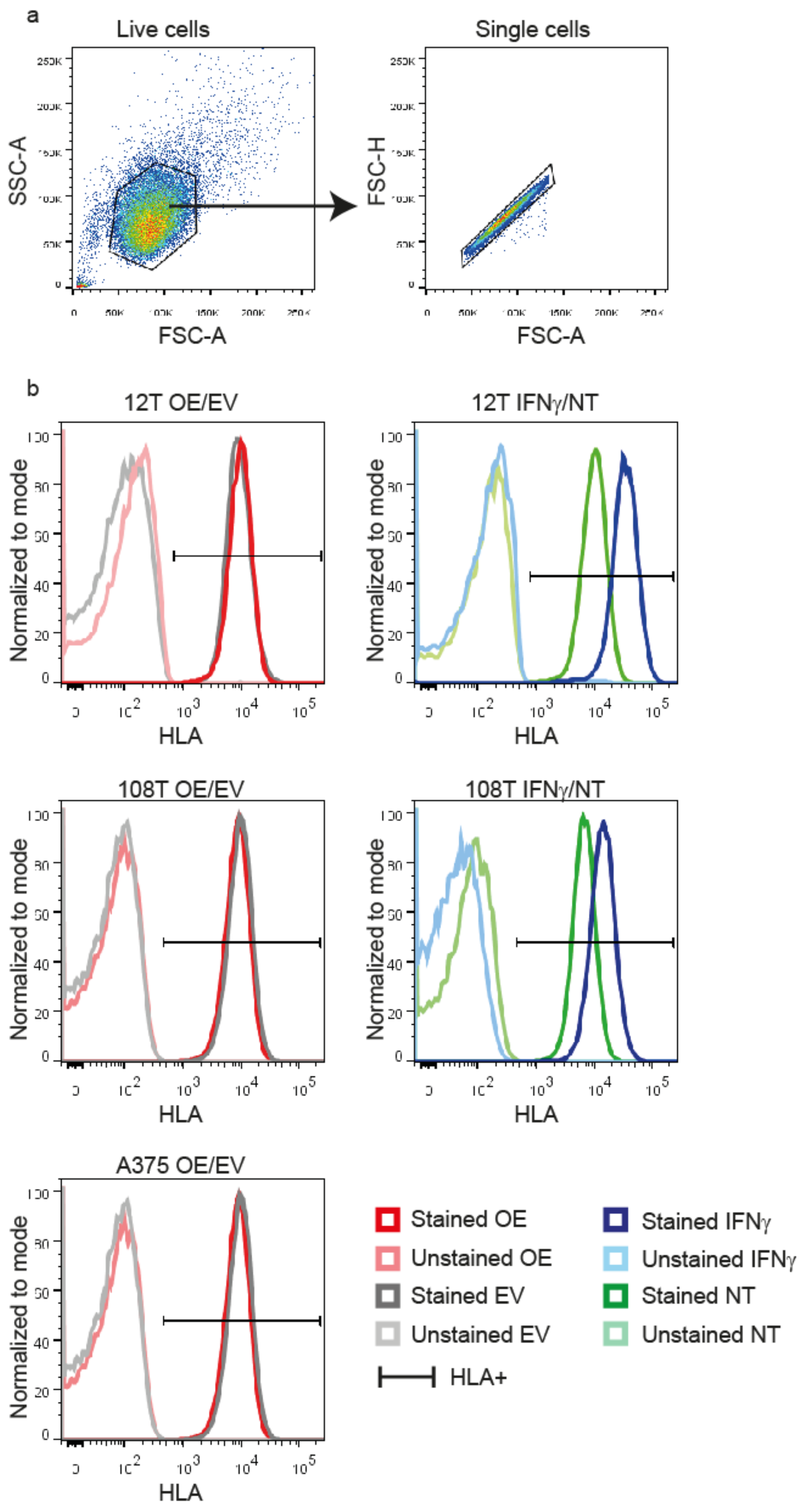
a
Live cells
Single cells
SSC-A
FSC-A
FSC-H
FSC-A
b
12T OE/EV
12T IFNγ/NT
108T OE/EV
108T IFNγ/NT
A375 OE/EV
Normalized to mode
HLA
Stained OE
Unstained OE
Stained EV
Unstained EV
HLA+
Stained IFNγ
Unstained IFNγ
Stained NT
Unstained NT

Supplementary Fig. 8. IFNγ treatment but not overexpression of immunoproteasome subunits increase HLA expression levels. Cells with overexpression of immunoproteasome subunit compare to empty vector control and IFNγ treated or non-treated cells were analyzed by flow cytometry to check the change in HLA expression. Each type of cells were stained and unstained with anti-HLA-A, B, C antibody. (a) Gating strategy for selecting live and single cells. (b) The gate marked in the figure indicate the area of positive HLA staining (HLA+) and showing that all cells express HLA-I on their surface. The shift in HLA staining in the samples that were treated with IFNγ show the increase in HLA-I expression, whereas there is no change after IP overexpression.

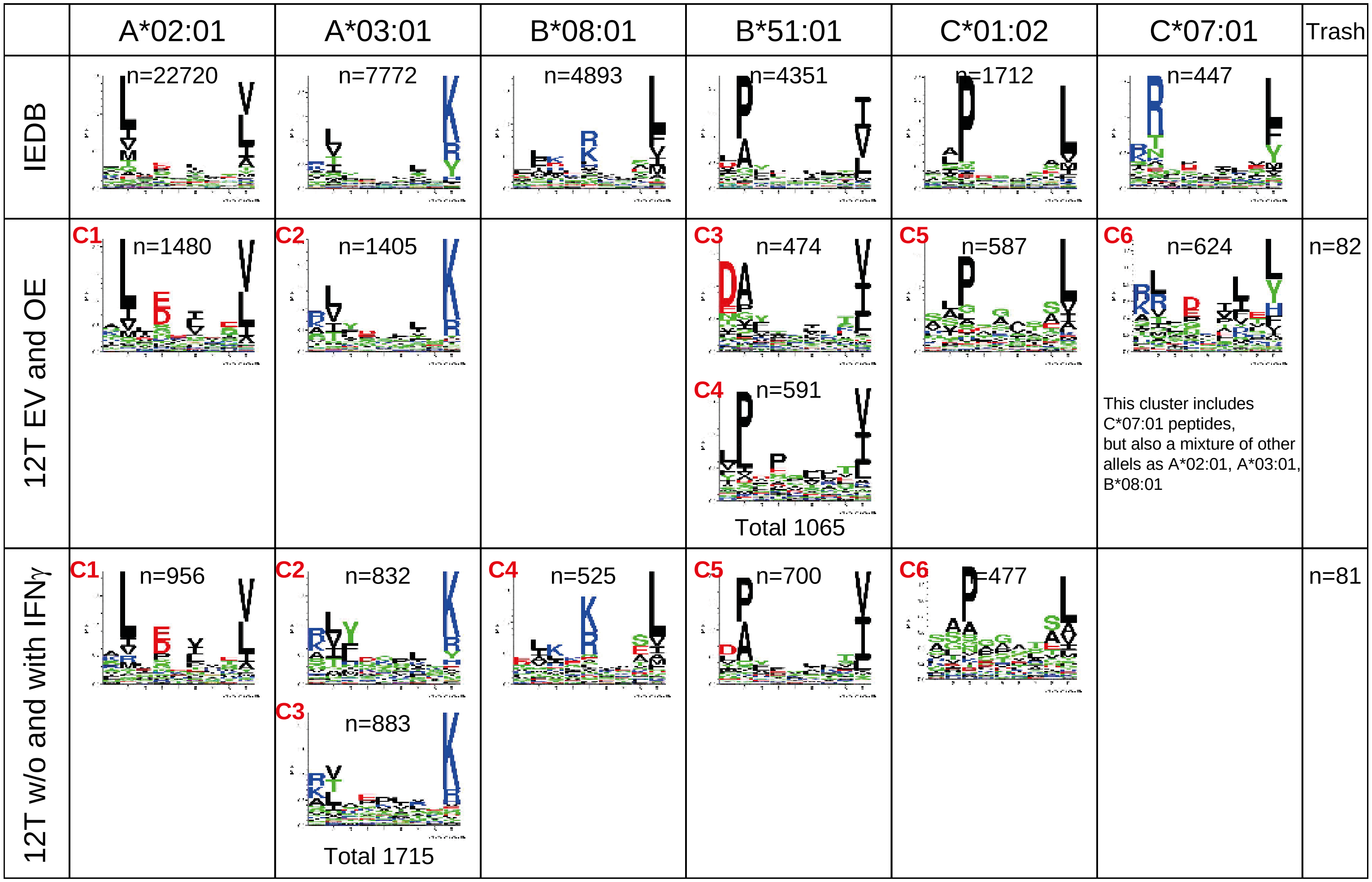

Supplementary Fig. 9. Clustering of identified HLA peptides in 12T cells to the patient HLA alleles. Gibbs clustering was used to cluster the peptides that were identified in the cells with immunoproteasome overexpression compare to empty vector and IFNγ treated and non-treated cells to six different clusters. The clusters show the motifs expected of the patient's HLA alleles. To find each allele motif, all peptides that match to this allele from the IEDB were clustered using Gibbs clustering. Each cluster is indicated by number in red ("CX", X=1-6). Trash column indicates the outlier peptides that were not clustered. Some clusters were grouped under the same allele motif, as these are the motifs that represent large number of peptides.

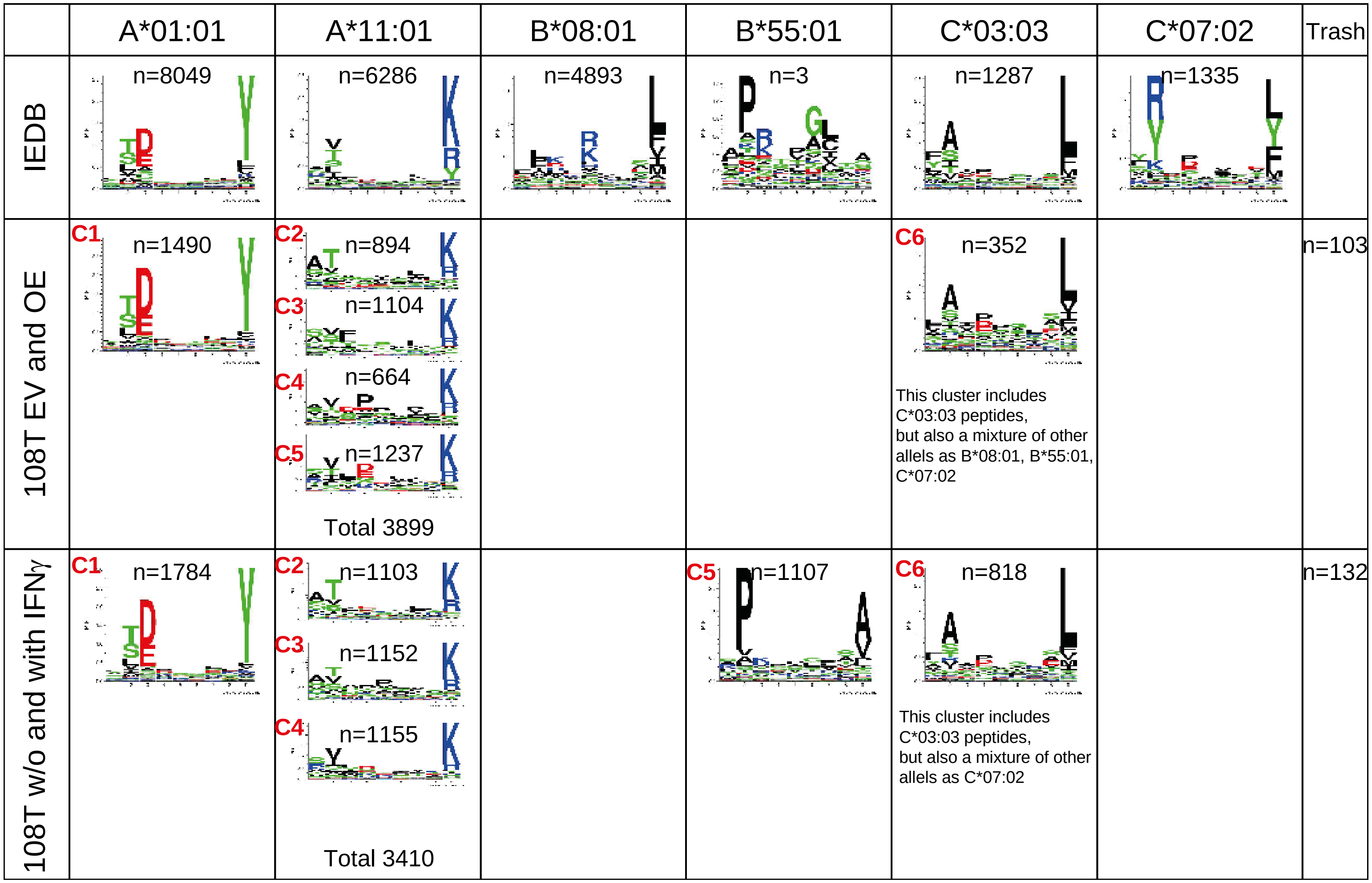

Supplementary Fig. 10. Clustering of identified HLA peptides in 108T cells to the patient HLA alleles. Gibbs clustering was used to cluster the peptides that were identified in the cells with immunoproteasome overexpression compare to empty vector and IFNγ treated and non-treated cells to six different clusters. The clusters show the motifs expected of the patient's HLA alleles. To find each allele motif, all peptides that match to this allele from the IEDB were clustered using Gibbs clustering. Each cluster is indicated by number in red ("CX", X=1-6). Trash column indicates the outlier peptides that were not clustered. Some clusters were grouped under the same allele motif, as these are the motifs that represent large number of peptides.

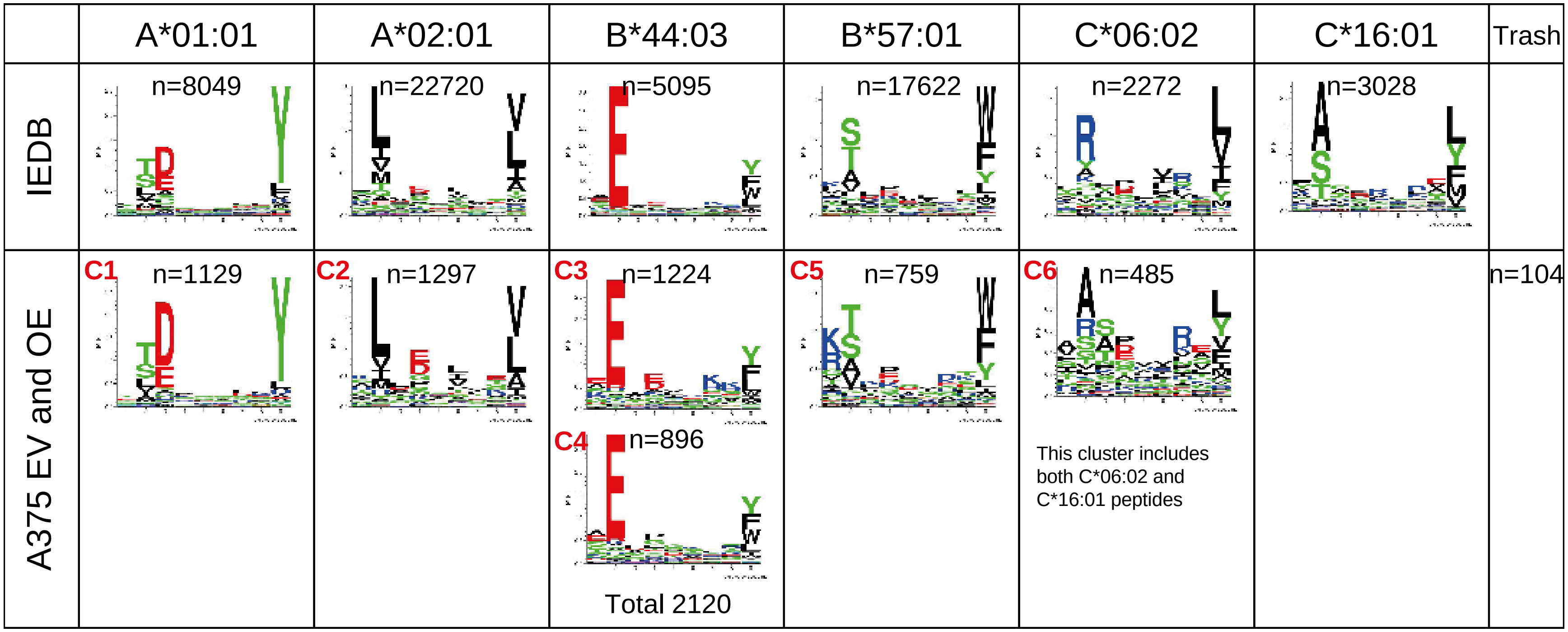

Supplementary Fig. 11. Clustering of identified HLA peptides in A375 cells to the patient HLA alleles. Gibbs clustering was used to cluster the peptides that were identified in the cells with immunoproteasome overexpression compare to empty vector and IFNγ treated and non-treated cells to six different clusters. The clusters show the motifs expected of the patient's HLA alleles. To find each allele motif, all peptides that match to this allele from the IEDB were clustered using Gibbs clustering. Each cluster is indicated by number in red ("CX", X=1-6). Trash column indicates the outlier peptides that were not clustered. Some clusters were grouped under the same allele motif, as these are the motifs that represent large number of peptides.

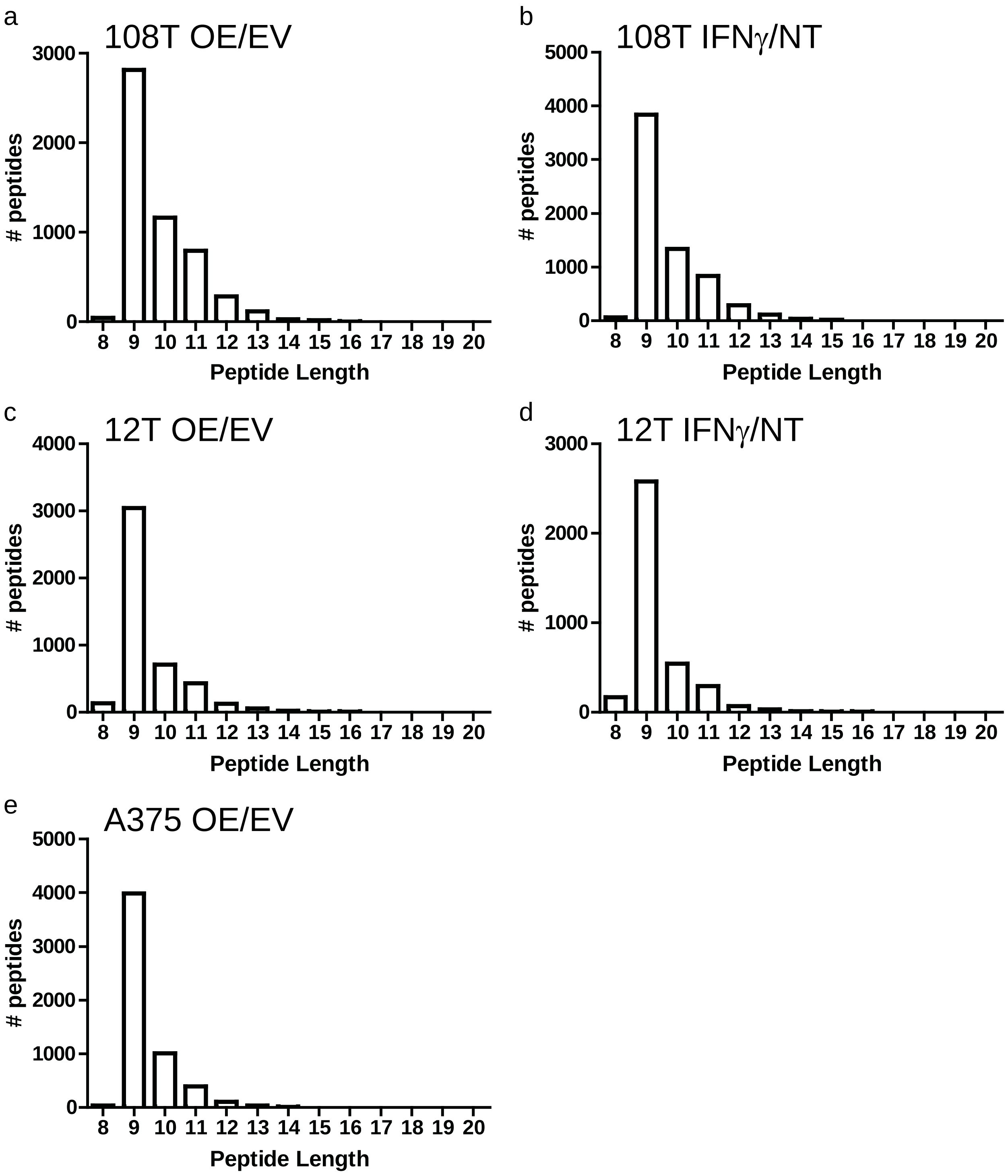

Supplementary Fig. 12. Length distribution of the identified peptides matched the expected distribution for HLA-I peptides.

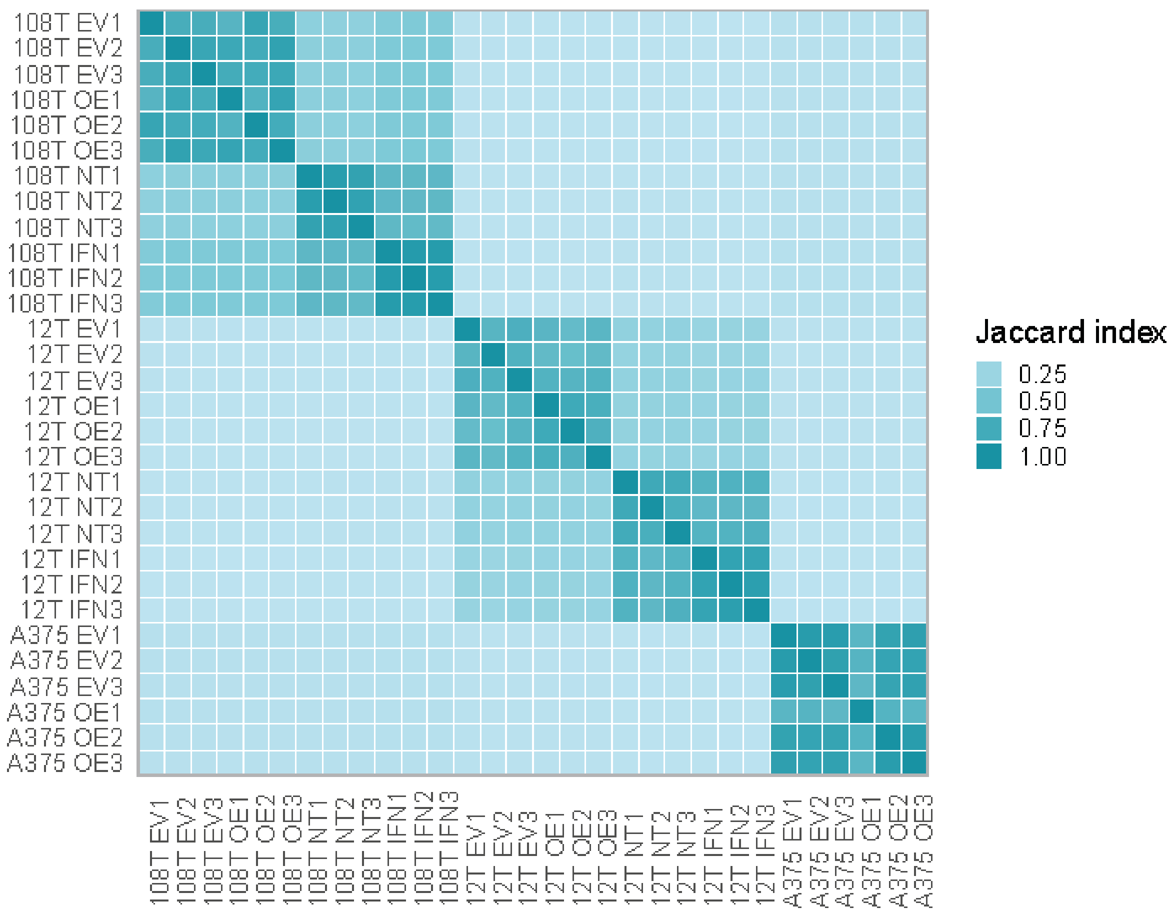

Supplementary Fig. 13. Biological replicates of HLA peptidomics experiments are similar. Jaccard index was calculated for each pair of samples. We observed high similarity between the replicates of the same experiment.

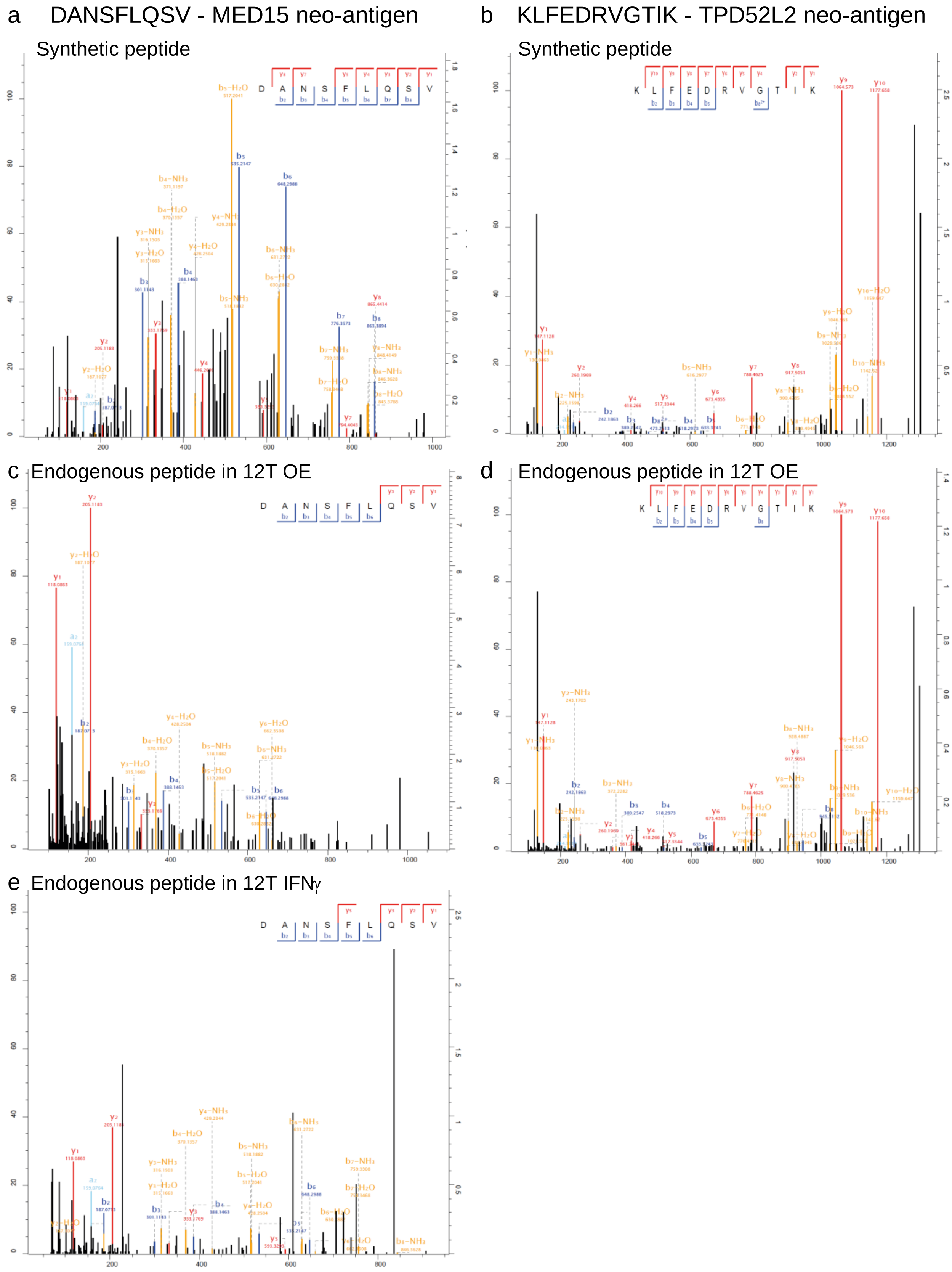
a DANSFLQSV - MED15 neo-antigen
Synthetic peptide
b KLFEDRVGTIK - TPD52L2 neo-antigen
Synthetic peptide
c Endogenous peptide in 12T OE
d Endogenous peptide in 12T OE
e Endogenous peptide in 12T IFNγ

Supplementary Fig. 14. Validation of neo-antigen identification using synthetic peptides. The MS/MS spectra of synthetic DANSFLQSV (a) or KLFEDRVGTIK (b) peptides were compared to the MS/MS spectra of the endogenous peptides in 12T OE cells (c, d) or IFNγ treated cells (e). The figures show representative figure of one of the MS/MS spectra, similar results were received in all samples in which the peptides were identified.

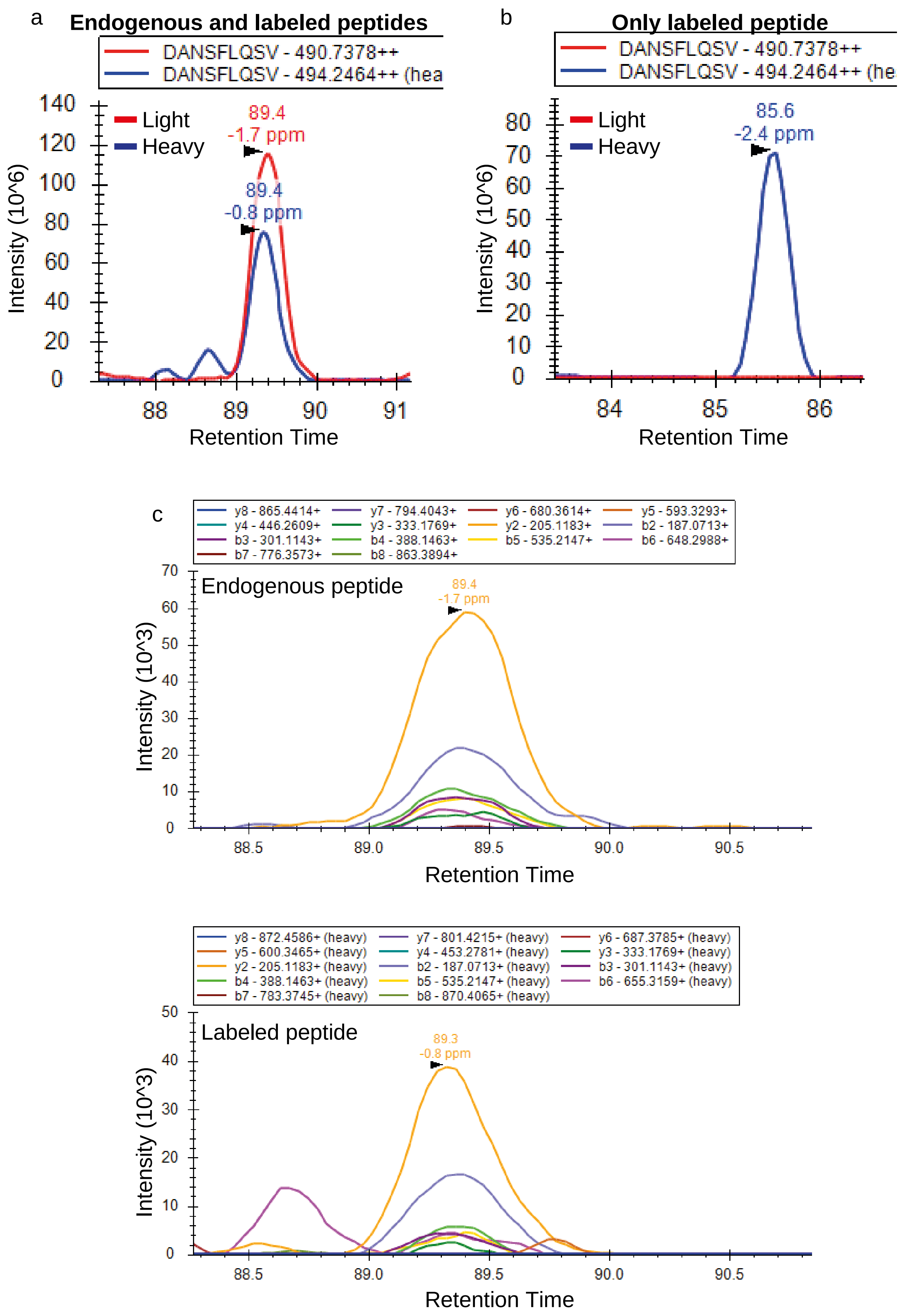
a
Endogenous and labeled peptides
DANSFLQSV - 490.7378++
DANSFLQSV - 494.2464++ (hea
Light
Heavy
89.4
-1.7 ppm
89.4
-0.8 ppm
Intensity (10^6)
Retention Time
b
Only labeled peptide
DANSFLQSV - 490.7378++
DANSFLQSV - 494.2464++ (he
Light
Heavy
85.6
-2.4 ppm
Intensity (10^6)
Retention Time
c
y8 - 865.4414+
y7 - 794.4043+
y6 - 680.3614+
y5 - 593.3293+
y4 - 446.2609+
y3 - 333.1769+
y2 - 205.1183+
b2 - 187.0713+
b3 - 301.1143+
b4 - 388.1463+
b5 - 535.2147+
b6 - 648.2988+
b7 - 776.3573+
b8 - 863.3894+
Endogenous peptide
89.4
-1.7 ppm
Intensity (10^3)
Retention Time
y8 - 872.4586+ (heavy)
y7 - 801.4215+ (heavy)
y6 - 687.3785+ (heavy)
y5 - 600.3465+ (heavy)
y4 - 453.2781+ (heavy)
y3 - 333.1769+ (heavy)
y2 - 205.1183+ (heavy)
b2 - 187.0713+ (heavy)
b3 - 301.1143+ (heavy)
b4 - 388.1463+ (heavy)
b5 - 535.2147+ (heavy)
b6 - 655.3159+ (heavy)
b7 - 783.3745+ (heavy)
b8 - 870.4065+ (heavy)
Labeled peptide
89.3
-0.8 ppm
Intensity (10^3)
Retention Time

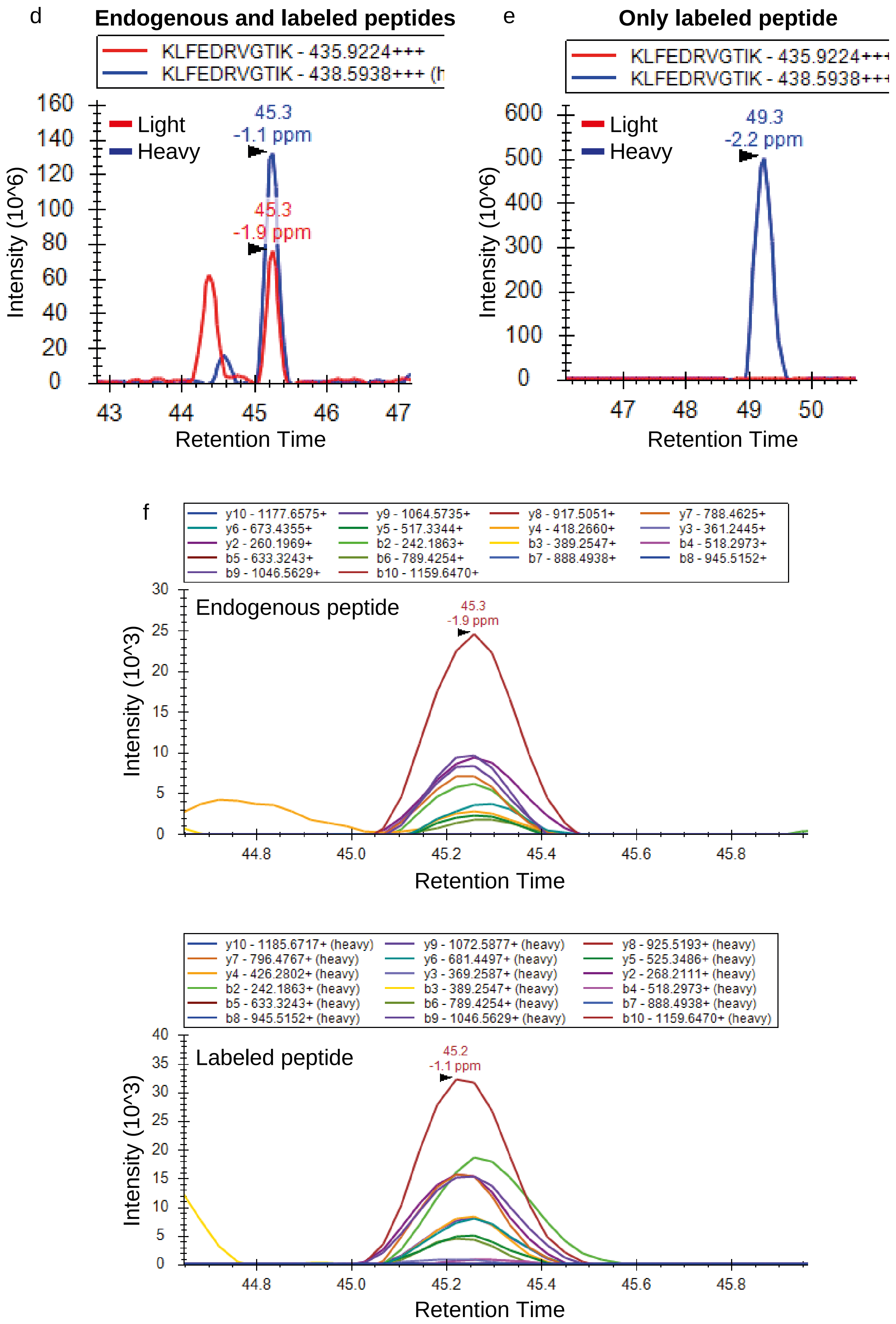
d
Endogenous and labeled peptides
KLFEDRVGTIK - 435.9224+++
KLFEDRVGTIK - 438.5938+++ (h
Light
Heavy
45.3
-1.1 ppm
45.3
-1.9 ppm
Intensity (10^6)
Retention Time
e
Only labeled peptide
KLFEDRVGTIK - 435.9224+++
KLFEDRVGTIK - 438.5938+++
Light
Heavy
49.3
-2.2 ppm
Intensity (10^6)
Retention Time
f
y10 - 1177.6575+
y9 - 1064.5735+
y8 - 917.5051+
y7 - 788.4625+
y6 - 673.4355+
y5 - 517.3344+
y4 - 418.2660+
y3 - 361.2445+
y2 - 260.1969+
b2 - 242.1863+
b3 - 389.2547+
b4 - 518.2973+
b5 - 633.3243+
b6 - 789.4254+
b7 - 888.4938+
b8 - 945.5152+
b9 - 1046.5629+
b10 - 1159.6470+
Endogenous peptide
45.3
-1.9 ppm
Intensity (10^3)
Retention Time
y10 - 1185.6717+ (heavy)
y9 - 1072.5877+ (heavy)
y8 - 925.5193+ (heavy)
y7 - 796.4767+ (heavy)
y6 - 681.4497+ (heavy)
y5 - 525.3486+ (heavy)
y4 - 426.2802+ (heavy)
y3 - 369.2587+ (heavy)
y2 - 268.2111+ (heavy)
b2 - 242.1863+ (heavy)
b3 - 389.2547+ (heavy)
b4 - 518.2973+ (heavy)
b5 - 633.3243+ (heavy)
b6 - 789.4254+ (heavy)
b7 - 888.4938+ (heavy)
b8 - 945.5152+ (heavy)
b9 - 1046.5629+ (heavy)
b10 - 1159.6470+ (heavy)
Labeled peptide
45.2
-1.1 ppm
Intensity (10^3)
Retention Time

Supplementary Fig. 15. Validation of neo-antigen identification using stable isotopically labeled synthetic peptides. DANSFLQSV (a) and KLFEDRVGTIK (d) stable isotopically labeled peptides were spiked to 12T OE sample and analyzed using mass spectrometry. The labeled and endogenous peptides are shown to be co-eluted. (b, e) The synthetic peptides were also analyzed alone to show that the synthetic heavy peptide is not contaminated with light peptide traces. (c, f) The MS2 fragment ions for the light and heavy peptide respectively. Data was analyzed using Skyline software. In a, d the blue line mark the labeled peptide and the red line mark the endogenous unlabeled peptide. The numbers indicate the peptides' measured retention time and mass error. The m/z of each peptide is indicated in the box and the "+" indicate the charge.

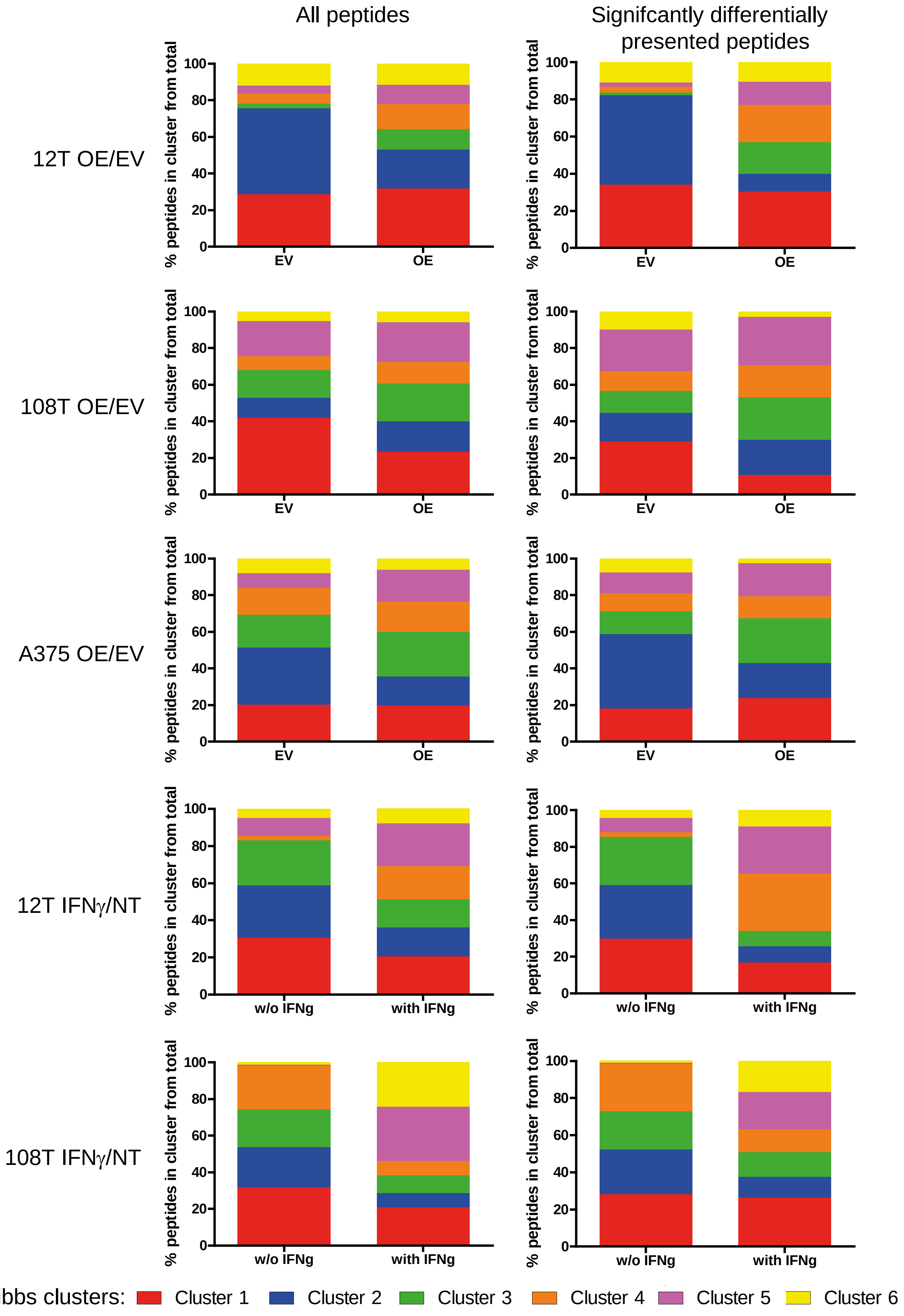

All peptides
Signifcantly differentially presented peptides
12T OE/EV
108T OE/EV
A375 OE/EV
12T IFNγ/NT
108T IFNγ/NT
% peptides in cluster from total
0
20
40
60
80
100
EV
OE
w/o IFNg
with IFNg
Gibbs clusters:
Cluster 1
Cluster 2
Cluster 3
Cluster 4
Cluster 5
Cluster 6

Supplementary Fig. 16. The change in the relative presentation of each cluster represent the changes in the alleles presentation after immunoproteasome overexpression or treatment with IFNγ. The percentage of each cluster representation by the differentially presented peptides was calculated for each cell line and condition. As expected, the peptides that were differentially presented by the cells with overexpression of immunoproteasome subunits were clustered more to alleles with chymotryptic-like motifs in their C-terminus (have A/F/I/L/M/V/Y amino acids in their c-terminus), and less to alleles with tryptic-like motifs (have K/R amino acids in their C-terminus). Peptides that were differentially presented by the cells that were treated with IFNγ show more representation by the HLA-B alleles.

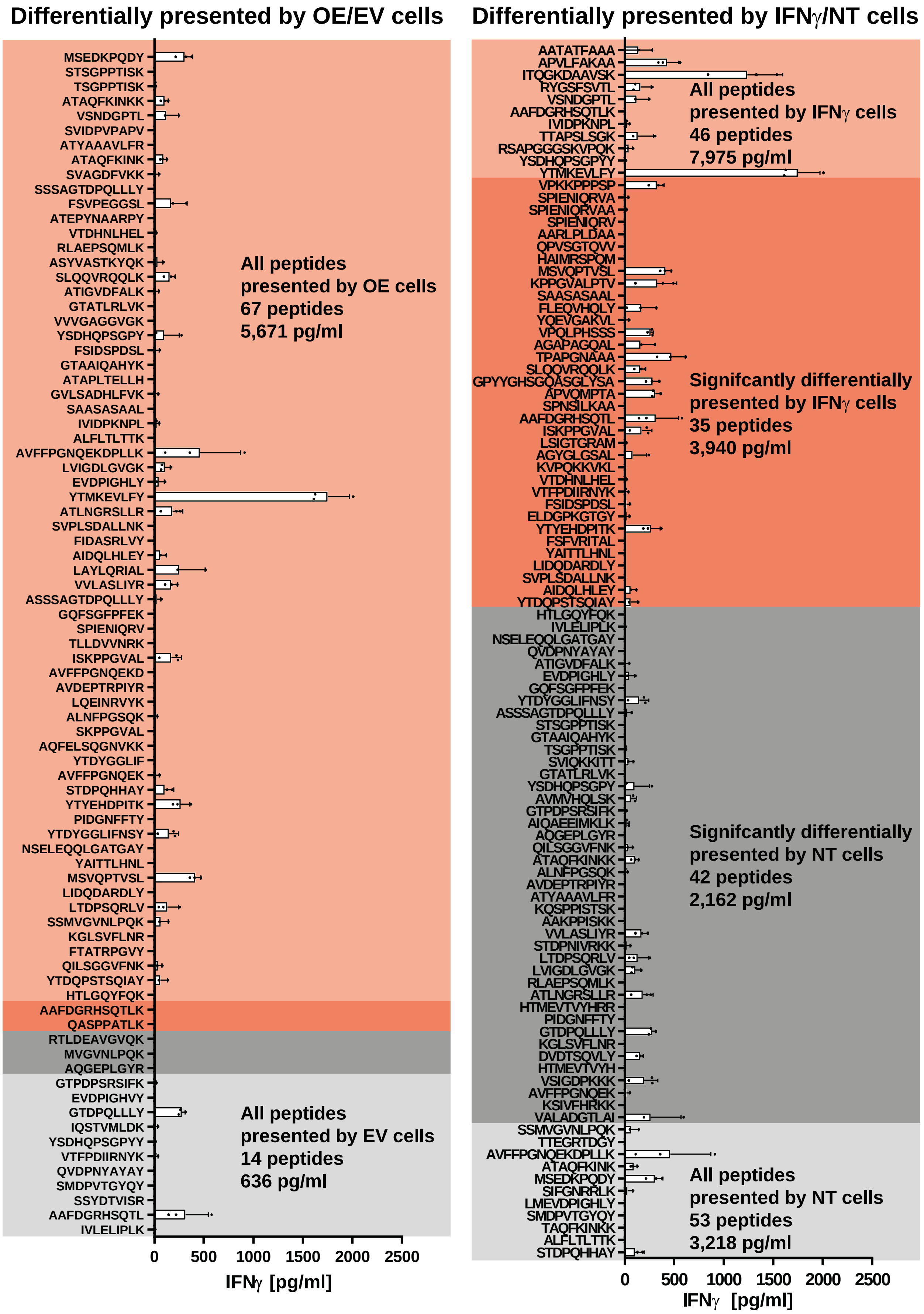

Differentially presented by OE/EV cells
Differentially presented by IFNγ/NT cells
All peptides presented by OE cells
67 peptides
5,671 pg/ml
All peptides presented by EV cells
14 peptides
636 pg/ml
All peptides presented by IFNγ cells
46 peptides
7,975 pg/ml
Signifcantly differentially presented by IFNγ cells
35 peptides
3,940 pg/ml
Signifcantly differentially presented by NT cells
42 peptides
2,162 pg/ml
All peptides presented by NT cells
53 peptides
3,218 pg/ml
IFNγ [pg/ml]
0
500
1000
1500
2000
2500

Supplementary Fig. 17. HLA peptide repertoire of 108T cells with overexpression of immunoproteasome subunits is more immunogenic compare to control repertoire. All peptides that their intensity fold change was greater or smaller than one, by cells with immunoproteasome overexpression compare to empty vector control or cells treated with IFNγ compare to non-treated cells, were tested for their ability to elicit an immune response by the autologous TILs. The reactivity of each peptide was measured from n=3 biological repeats per each peptide and represented as mean ± SD. The sum reactivity of each group of peptides, and the number of peptides in each group is indicated. Red and gray areas represent all peptides that they intensity fold change was greater or smaller than one by the OE/IFNγ cells and EV/NT cells respectively (including the significantly changed peptides). Dark red and dark gray areas represent the peptides that were significantly differentially presented by the IFNγ cells and NT cells respectively. As the number of significantly differentially presented peptides in the OE/EV is low and all these peptides are non-immunogenic, their summed reactivity is not indicated in the figure. For each condition the sum reactivity was calculated for all peptides and for the ones that were significantly differentially presented.

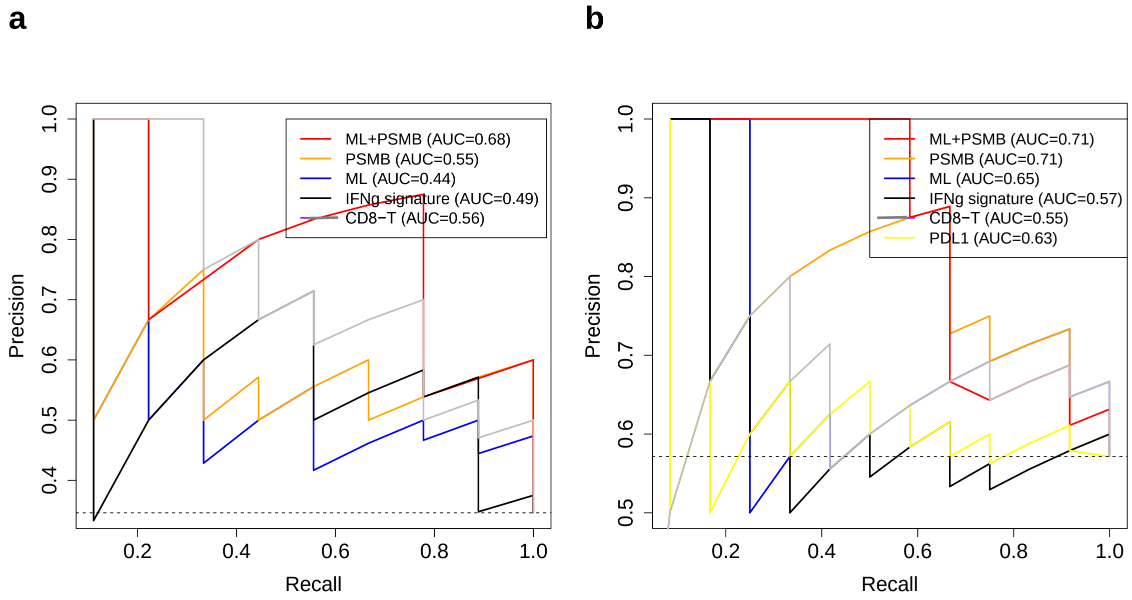

Supplementary Fig. 18. IP subunits predicts the success of immune checkpoint therapy in high purity tumors. The precision-recall curves show that (a) IP expression itself shows the highest prediction accuracy for anti-PD1 therapy followed by mutational load, PDL1 expression, IFNγ signature, and CD8+ T-cell abundance. (b) IP expression shows the highest prediction accuracy for anti-CTLA4 therapy when combined with mutational load, followed by CD8+ T-cell abundance, IFNγ signature, and mutational load.
