# Supplementary material for: Immunoproteasome expression is associated with better prognosis and response to checkpoint therapies in melanoma
Source: Nat Commun. 2020 Feb 14;11:896. doi: 10.1038/s41467-020-14639-9 (PMC7021791; doi:10.1038/s41467-020-14639-9)
Supplement: Supplementary file 4 — Reporting Summary [file 41467_2020_14639_MOESM4_ESM.pdf]

natureresearch

Corresponding author(s): Prof. Yardena Samuels

Reporting Summary

Nature Research wishes to improve the reproducibility of the work that we publish. This form provides structure for consistency and transparency in reporting. For further information on Nature Research policies, see Authors & Referees and the Editorial Policy Checklist.

Statistical parameters

When statistical analyses are reported, confirm that the following items are present in the relevant location (e.g. figure legend, table legend, main text, or Methods section).

n/a	Confirmed	
☐	☒	The exact sample size (n) for each experimental group/condition, given as a discrete number and unit of measurement
☐	☒	An indication of whether measurements were taken from distinct samples or whether the same sample was measured repeatedly
☐	☒	The statistical test(s) used AND whether they are one- or two-sided<br>*Only common tests should be described solely by name; describe more complex techniques in the Methods section.*
☐	☒	A description of all covariates tested
☐	☒	A description of any assumptions or corrections, such as tests of normality and adjustment for multiple comparisons
☐	☒	A full description of the statistics including central tendency (e.g. means) or other basic estimates (e.g. regression coefficient) AND variation (e.g. standard deviation) or associated estimates of uncertainty (e.g. confidence intervals)
☐	☒	For null hypothesis testing, the test statistic (e.g. F, t, r) with confidence intervals, effect sizes, degrees of freedom and P value noted<br>*Give P values as exact values whenever suitable.*
☐	☒	For Bayesian analysis, information on the choice of priors and Markov chain Monte Carlo settings
☐	☒	For hierarchical and complex designs, identification of the appropriate level for tests and full reporting of outcomes
☐	☒	Estimates of effect sizes (e.g. Cohen's d, Pearson's r), indicating how they were calculated
☐	☒	Clearly defined error bars<br>*State explicitly what error bars represent (e.g. SD, SE, CI)*

Our web collection on statistics for biologists may be useful.

Software and code

Policy information about availability of computer code

Data collection	Not relevant
Data analysis	MaxQuant version 1.5.3.8<br>NetMHCpan version 4.0<br>Perseus computational platform version 1.6.6.0

For manuscripts utilizing custom algorithms or software that are central to the research but not yet described in published literature, software must be made available to editors/reviewers upon request. We strongly encourage code deposition in a community repository (e.g. GitHub). See the Nature Research guidelines for submitting code & software for further information.

Data

Policy information about availability of data

All manuscripts must include a data availability statement. This statement should provide the following information, where applicable:

- Accession codes, unique identifiers, or web links for publicly available datasets
- A list of figures that have associated raw data
- A description of any restrictions on data availability

TCGA data was downloaded from the Genomic Data Common (GDC) TCGA data portal, https://portal.gdc.cancer.gov, described in methods section.

GTEX samples from UCSC Xena browser available at https://toil.xenahubs.net/download/TcgaTargetGtex_RSEM_Hugo_norm_count.gz.
Predicting the effectiveness of immune checkpoint inhibitors therapy was done using two different published melanoma cohorts from Hugo et al., 2016 and Van allen et al., 2015. Described in methods section
Whole exome sequencing (WES) of 108T and 12T is available in dbSNP under accession 1062266.
GibbsCluster 2.0 server (www.cbs.dtu.dk/services/GibbsCluster)
Immune epitope database (IEDB, www.iedb.org)
Seq2Logo 2.0 (http://www.cbs.dtu.dk/biotools/Seq2Logo)
HLA peptidomics data have been deposited to the ProteomeXchange Consortium via the PRIDE partner repository with the dataset identifier PXD015957.

Field-specific reporting

Please select the best fit for your research. If you are not sure, read the appropriate sections before making your selection.

☒ Life sciences ☐ Behavioural & social sciences ☐ Ecological, evolutionary & environmental sciences

For a reference copy of the document with all sections, see nature.com/authors/policies/ReportingSummary-flat.pdf

Life sciences study design

All studies must disclose on these points even when the disclosure is negative.

Sample size	Not relevant
Data exclusions	Not relevant
Replication	All experiments were done in three replicates
Randomization	Not relevant
Blinding	Not relevant

Reporting for specific materials, systems and methods

Materials & experimental systems

n/a	Involved in the study	
☒	☐	Unique biological materials
☐	☒	Antibodies
☐	☒	Eukaryotic cell lines
☒	☐	Palaeontology
☒	☐	Animals and other organisms
☒	☐	Human research participants

Methods

n/a	Involved in the study	
☒	☐	ChIP-seq
☐	☒	Flow cytometry
☒	☐	MRI-based neuroimaging

Antibodies

Antibodies used	anti-PSMB8: cat no 13726, from Cell Signaling<br>anti-PSMB9: cat no 3328, from Abcam<br>anti-GAPDH: cat no MAB374, from Millipore<br>PE/Cy7 anti-HLA-A, B, C (W6/32) antibody, cat no 311429, from Biolegend
Validation	Found in manufacture's website

Eukaryotic cell lines

Policy information about cell lines

Cell line source(s)	12T and 108T are received from the NIH (in methods section) and A375 is from ATCC

Authentication	Cells were authenticated by Finger printing with STR profiling (Panel: PowerPlex_16_5Nov142UAGC, Size: GS500 x35 x50 x250, Analysis Type: Fragment (Animal), Software Package: SoftGenetics GeneMarker 1.85)
Mycoplasma contamination	All cell lines were tested regularly and were found negative for mycoplasma contamination (EZ-PCR Mycoplasma Kit, Biological Industries)
Commonly misidentified lines (See ICLAC register)	Not relevant

Flow Cytometry

Plots

Confirm that:

- [x] The axis labels state the marker and fluorochrome used (e.g. CD4-FITC).
- [x] The axis scales are clearly visible. Include numbers along axes only for bottom left plot of group (a 'group' is an analysis of identical markers).
- [x] All plots are contour plots with outliers or pseudocolor plots.
- [x] A numerical value for number of cells or percentage (with statistics) is provided.

Methodology

Sample preparation	5*10^6 12T and 108T cells with immunoproteasome overexpression and empty vector control, and treated or non-treated with IFNg were collected and washed with PBS. Then cells were incubated with PE/Cy7 anti-HLA-A, B, C (W6/32) antibody (311429, Biolegend) for 30 minutes on ice. Cells were later washed twice with PBS and analysed using BD LSR II flow cytometer (BD Biosciences)
Instrument	BD LSR II flow cytometer (BD Biosciences)
Software	Data was analyzed using the FlowJo software
Cell population abundance	Unstained population was used to gate the positively stained population
Gating strategy	Cells were first gated for live single cells, and then unstained population was used to gate the positively stained population. Stained samples were compared for their HLA staining to define HLA expression. Gating is described in Supplementary figure 8

- [x] Tick this box to confirm that a figure exemplifying the gating strategy is provided in the Supplementary Information.
