# Supplementary material for: Immunoproteasome expression is associated with better prognosis and response to checkpoint therapies in melanoma
Source: Nat Commun. 2020 Feb 14;11:896. doi: 10.1038/s41467-020-14639-9 (PMC7021791; doi:10.1038/s41467-020-14639-9)
Supplement: Supplementary file 5 — Source Data [file 41467_2020_14639_MOESM5_ESM.zip › Uncropped western blots images.pptx]

## Slide 1
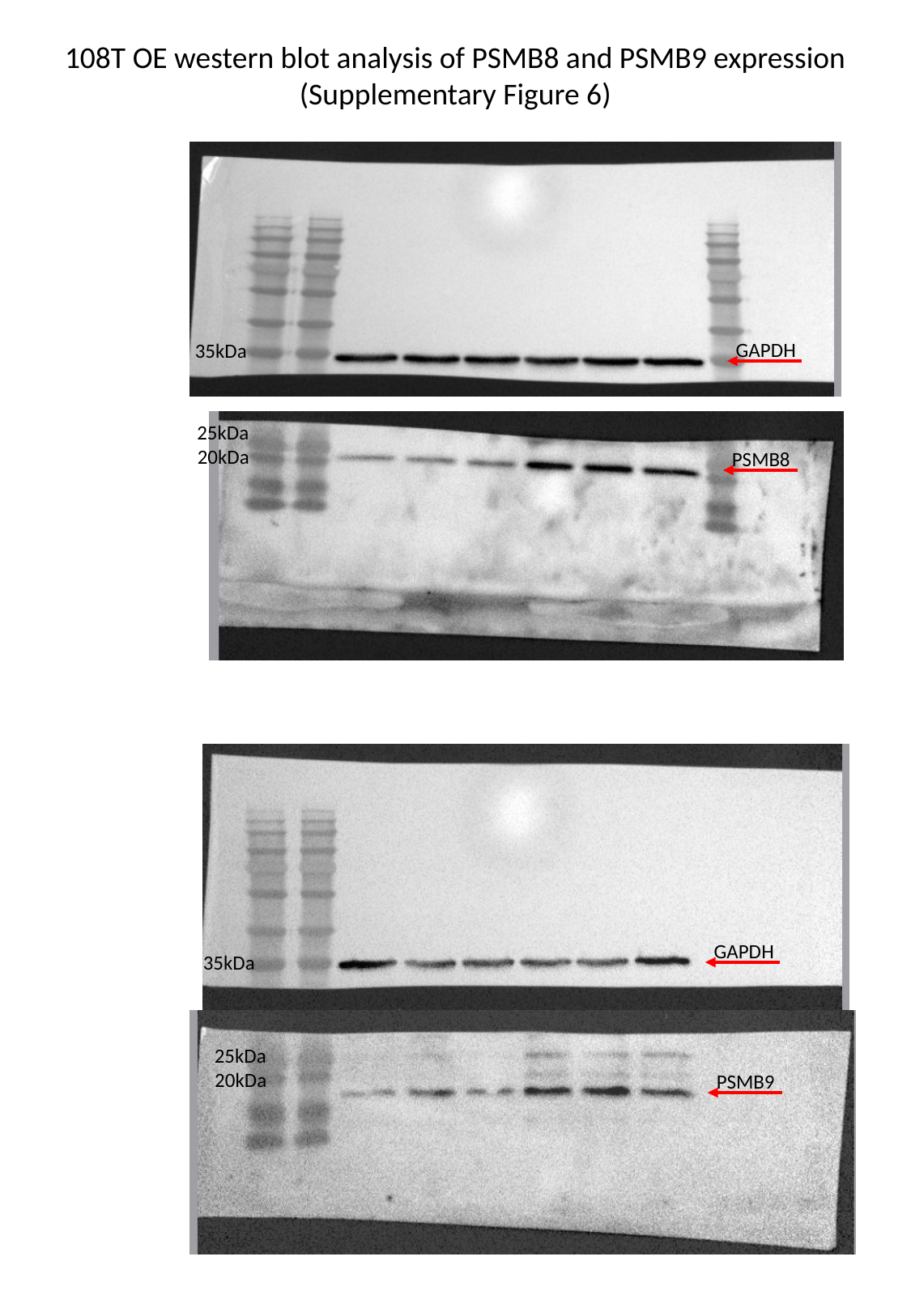

108T OE western blot analysis of PSMB8 and PSMB9 expression
(Supplementary Figure 6)
GAPDH
35kDa
25kDa
20kDa
PSMB8
GAPDH
35kDa
25kDa
20kDa
PSMB9

## Slide 2
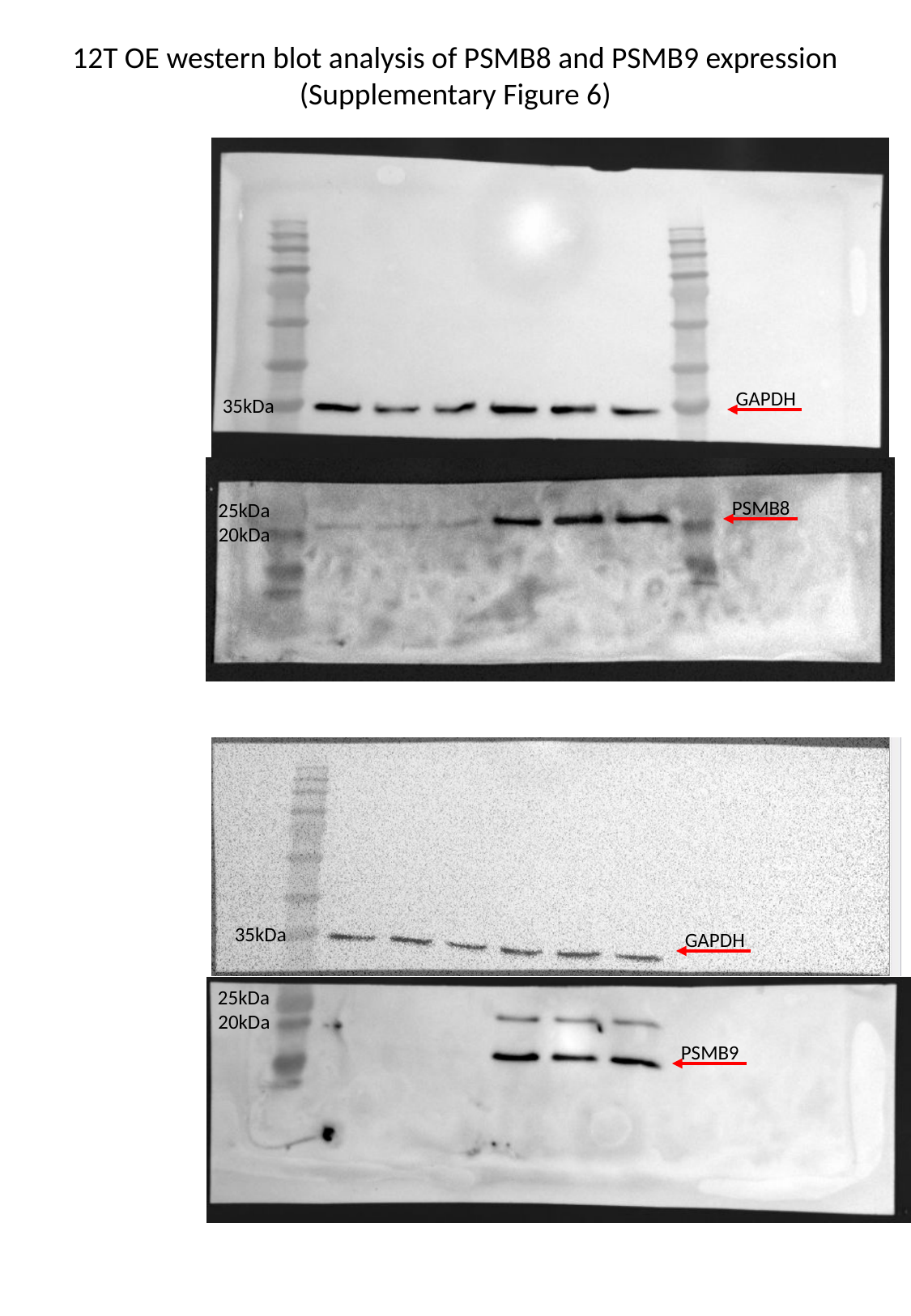

12T OE western blot analysis of PSMB8 and PSMB9 expression
(Supplementary Figure 6)
GAPDH
35kDa
PSMB8
25kDa
20kDa
35kDa
GAPDH
25kDa
20kDa
PSMB9

## Slide 3
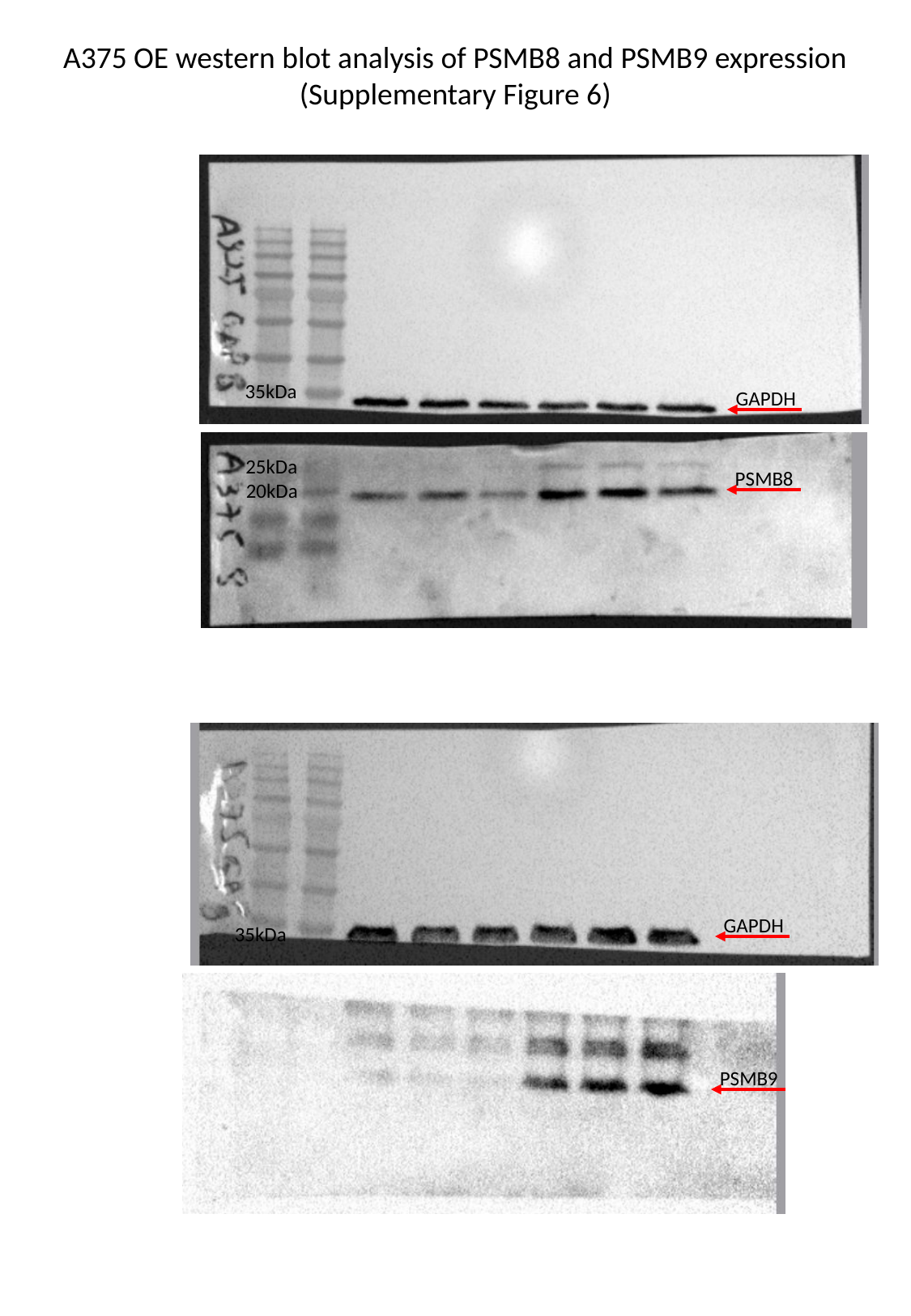

A375 OE western blot analysis of PSMB8 and PSMB9 expression
(Supplementary Figure 6)
35kDa
GAPDH
25kDa
PSMB8
20kDa
GAPDH
35kDa
PSMB9

## Slide 4
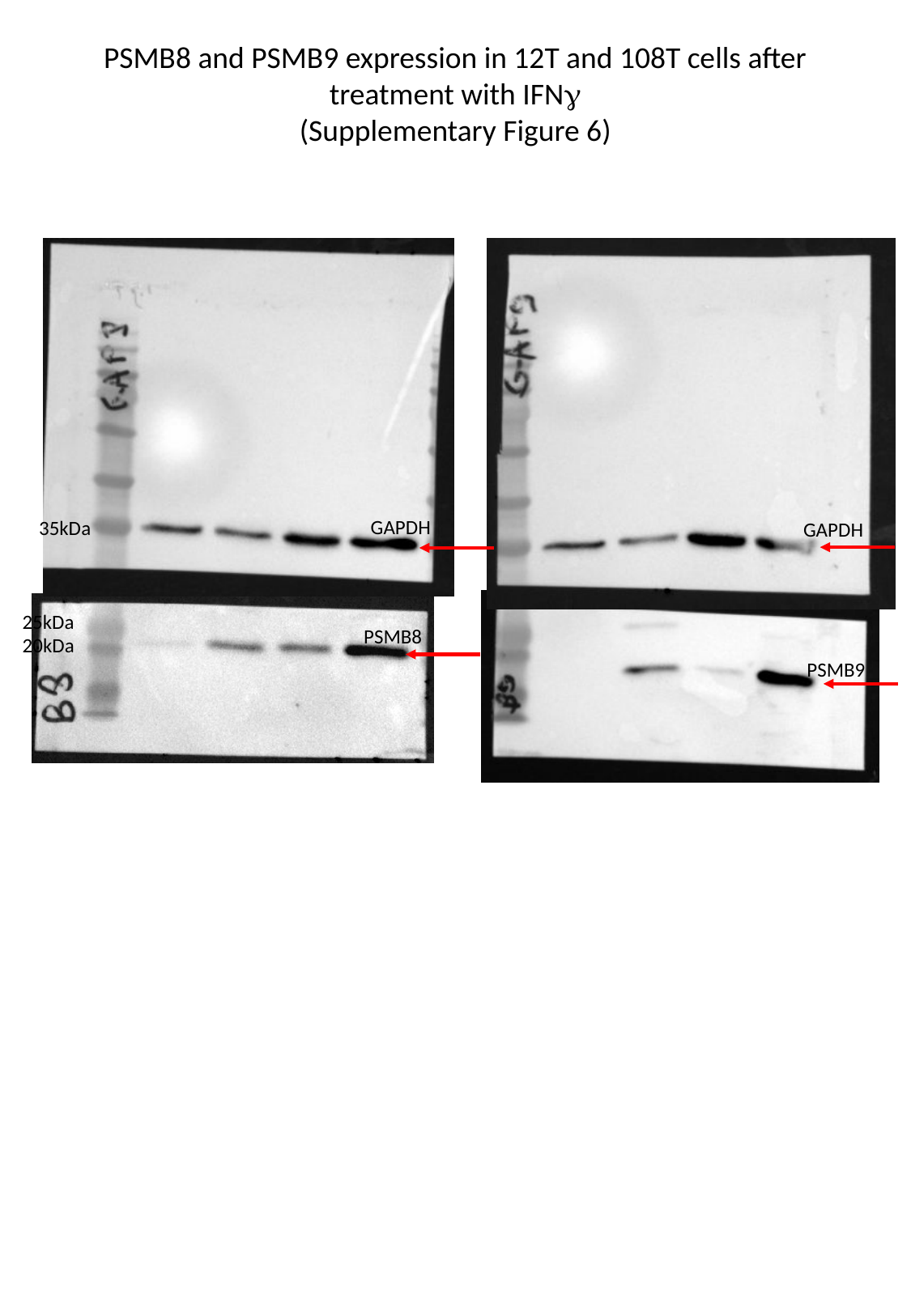

PSMB8 and PSMB9 expression in 12T and 108T cells after treatment with IFNg
(Supplementary Figure 6)
GAPDH
35kDa
GAPDH
25kDa
PSMB8
20kDa
PSMB9
